# 基于微型质谱仪的实验教学实践创新：从质谱原理到测试应用

**DOI:** 10.3724/SP.J.1123.2025.06022

**Published:** 2026-07-08

**Authors:** Lan LI, Jie HONG, Xinhui YU, Peifeng GAO, Yanbing ZHAI

**Affiliations:** 1. 北京理工大学资产与实验室管理处，北京 100081; 1. Office of National Assets & Laboratory Management，Beijing Institute of Technology，Beijing 100081，China; 2. 昆山聂尔精密仪器有限公司，江苏 苏州 215316; 2. Kunshan Nier Precision Instrumentation Inc. ，Suzhou 215316，China; 3. 北京理工大学医学技术学院，北京 100081; 3. School of Medical Technology，Beijing Institute of Technology，Beijing 100081，China

**Keywords:** 实验教学, 微型质谱仪, 线性离子阱, 血药检测, 谱库筛查, experimental teaching, miniature mass spectrometer, linear ion trap, blood drug determination, library screening

## Abstract

传统质谱教学因商业化质谱仪体积庞大、操作复杂、成本高昂，普遍存在“重理论、轻实践”的困境。微型质谱仪凭借体积小、操作简便成为理想的替代教学工具。它采用大气压电离源降低样品前处理要求；通过线性离子阱实现质谱仪体积的缩小但又不损失功能；结合微型离子漏斗与大气压连续进样接口保障了分析的高效稳定。微型质谱仪具备串联质谱（MS *^n^* ）分析功能，足以满足教学中有机物结构解析与复杂样品快速检测需求。本课程以提升学生知识、能力、素养的教学目标为导向，通过仪器拆解、参数优化和实际应用案例，设计了以下教学实验：通过仪器模块化拆解认知结构，结合质量轴校准、全扫描分析，帮助学生掌握仪器基本操作；选择离子扫描和串联质谱分析，深度训练学生质谱数据解析与结构推导能力；融入西药成分确证、血药检测和中药多组分筛查等实际案例，培养学生运用质谱解决复杂实际问题的科研素养与创新能力。通过构建高效合理的教学计划，推动该实验教学课程在计划内教学和开放实验教学等课程中稳步落地，并通过教学反思和后续课程的迭代优化，强化学生的科研思维与实践能力，为其适应现代科研与产业需求奠定了坚实基础，也显著提升了仪器分析课程的实践性与创新性。

## Abstract (en)

Mass spectrometer is an important component of the instrumental analysis courses in higher education. However， conventional commercial mass spectrometers usually lead to an imbalance between theory and practice in teaching， because of their characteristics of large size， high price and complicated operations， which makes it difficult to establish a direct link between instrument structure and detection principles. The miniature mass spectrometer shares the same core principles as commercial mass spectrometers. Equipped with an atmospheric pressure ionization （API） source， it significantly reduces sample preparation requirements， and enables rapid analysis of complex samples. The unique capability of the linear ion trap mass analyzer to scan， store， and eject ions enables a dramatic reduction in instrument size. The miniature ion funnel transmission technology and atmospheric pressure continuous sampling interface ensure high repeatability and rapid analysis， allowing compatibility with various atmospheric pressure ion sources for diverse sample types. In terms of performance， the miniature mass spectrometer has a mass-to-charge （*m/z*） scan range of 50-2 000， and supports tandem mass spectrometry （MS*ⁿ*）， enabling structural analysis of common organic compounds and rapid testing of complex samples. Therefore， the miniature mass spectrometer becomes an ideal tool for experimental teaching. This teaching course has developed a “three-dimensional integrated” teaching system aimed at enhancing students’ knowledge， abilities， and literacy. Firstly， students disassemble key components of the instrument such as the ion source， ion funnel and trap to understand how they work together to achieve sample ionization， transmission， and acquisition. Mass calibration and full scan test help students master the basic operation. By adjusting the radio frequency （RF） voltage parameters， students could observe the impact of different parameters on the experimental results. Secondly， selected ion monitoring and tandem MS analysis help enhance students’ data analysis skills and further develop their abilities in structural confirmation of compounds with MS. Lastly， pharmaceutical ingredient analysis， blood drug testing and multi-component screening of traditional Chinese medicine comprehensively demonstrate the wide applications of MS in scientific research and daily life， aiming to cultivate students’ research thinking and problem-solving abilities. The gradient experiments （from basic calibration to advanced MS *^n^* ） combined with practical applications have effectively driven the implementation of the offline teaching course. The suggested experimental class hours are 12， covering pre-class preparation， in-class hands-on practice， data acquisition， and post-class summary. Practice has demonstrated that this integrated “structure-principle-application” teaching model effectively bridges the knowledge chain from theoretical learning to practical application， and significantly enhances students’ innovative thinking and ability to solve complex analytical problems， better preparing them to meet the requirements of modern scientific research and industrial applications. Additionally， instructional reflections and feasible teaching optimizations are necessary based on the teaching effects and feedback. This comprehensive learning experience provides students with invaluable inspiration and guidance， and lays a solid foundation for them to enter the field of scientific research， having successfully cultivated talent with practical abilities and innovation literacy， and achieving the intended teaching objectives.

质谱仪作为一种可精确测定化合物质荷比（*m/z*）的精密仪器，在食品^［1-3］^、药物^［4，5］^、环境^［6-8］^、临床医学^［9-11］^等领域发挥着不可替代的作用，也是生物、化学、材料、环境等学科课程学习和实验必不可少的仪器设备。然而在当前高校教育体系中，质谱仪实验教学正面临着技术与教学范式显著脱节的问题：实验室普遍配置的大型进口质谱仪不仅体积庞大、构造复杂，且购置与维护成本高昂、操作步骤繁复，这些因素共同导致教学过程中长期存在“重理论、轻实践”的困境。现有教学模式多采用课件演示与速成型验证实验相结合的方式^［12-15］^，这种碎片化的教学设计不仅难以建立仪器构造与检测原理间的直观联系，更因操作空间的限制无法充分展现质谱技术的核心优势，最终导致学生对质谱技术的认知停留在抽象概念层面，难以高效理解和消化质谱仪的关键原理，进而难以激发其科研探索热情。

在质谱分析中，针对未知样品选择适宜的数据采集模式，是获得信息丰富、可靠质谱数据的基础与关键环节。由于化合物结构差异显著，学生在实际分析中常面临模式选择的困难。季铵盐在溶液中易于解离为阴阳离子，在电喷雾离子源中具有较高的电离效率，因此可作为代表性化合物用于多种谱图采集的教学示例，帮助学生理解不同模式的适用范围。此外，在实践应用层面，血液中药物检测与筛查是体外诊断和临床医学中一项成熟且广泛使用的分析技术。掌握该技术的原理与方法，有助于提升学生将基础知识应用于实际科学问题的能力。近年来，微型质谱仪（miniature mass spectrometer， mini-MS）关键技术的突破以及系统整机的商品化为分析仪器教学改革提供了新契机。微型质谱仪在继承传统质谱核心原理和分析性能的基础上^［16-18］^，通过模块化设计实现了革命性突破：其紧凑型结构（通常<30 kg）不仅支持现场快速检测^［19-21］^，更因其开放式架构显著提升了教学适用性。特别值得关注的是，国产微型质谱仪在常压电离、自主电路设计、智能软件系统等关键技术的突破^［22-24］^，不仅打破了进口仪器的技术垄断，其便于拆装维护的结构特点更为实验教学提供了理想的实践平台。Xu等^［25］^基于直流诱导电喷雾离子化（direct current-induced electrospray ionization， DC-iESI）技术设计教学实验，学生使用纳升级电喷雾喷针，直接从柑橘果皮的油腺和有色皮层采集微量液体，实现了对这两个微区成分的原位质谱分析，以此加深学生对原位质谱分析的理解及应用。Zhang等^［26］^在本科生教学实验中利用微型质谱仪对盐酸小檗碱纯化产物进行快速定性和定量分析，为质谱分析纳入常规本科教学提供了范例。对比传统教学设备的局限性，微型质谱仪在实验教学领域展现出五大适配优势：（1）可视化机械结构助力原理认知；（2）模块化设计支持动手组装实践；（3）智能化软件降低操作门槛；（4）快速检测功能增强应用感知；（5）自主研发体系培养工程思维。

本课程创新性地构建了“三维一体”的教学体系：在知识维度，通过仪器拆解展示电离源、质量分析器等核心组件的构造组装；在操作维度，设计梯度式实验项目（参数优化、模式切换、定性分析）培养仪器操控能力；在应用维度，开发药品成分鉴定、血药快速筛查、中药真伪鉴别等现实场景分析案例。该体系突出多学科交叉特色，融合机械、电子、化学等学科知识，培养学生从原理认知到实践应用的综合能力。整合教学目标、教学模式与教学内容三大核心模块，课程详细架构如图1所示。

**图1:**
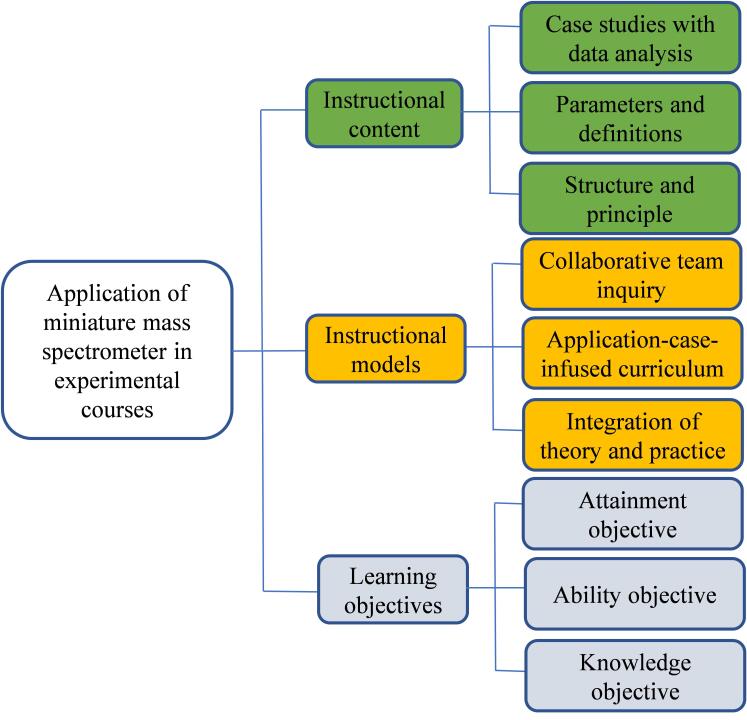
实验教学课程架构

## 1 实验部分

### 1.1 微型质谱仪构造与原理

微型质谱仪主机构造及其可拆解模块化部件实物图见图2，仪器主要由真空系统、一次性采样进样系统、电喷雾离子源、离子传输组件、线性离子阱质量分析器构成，并实现如下功能。

**图2:**
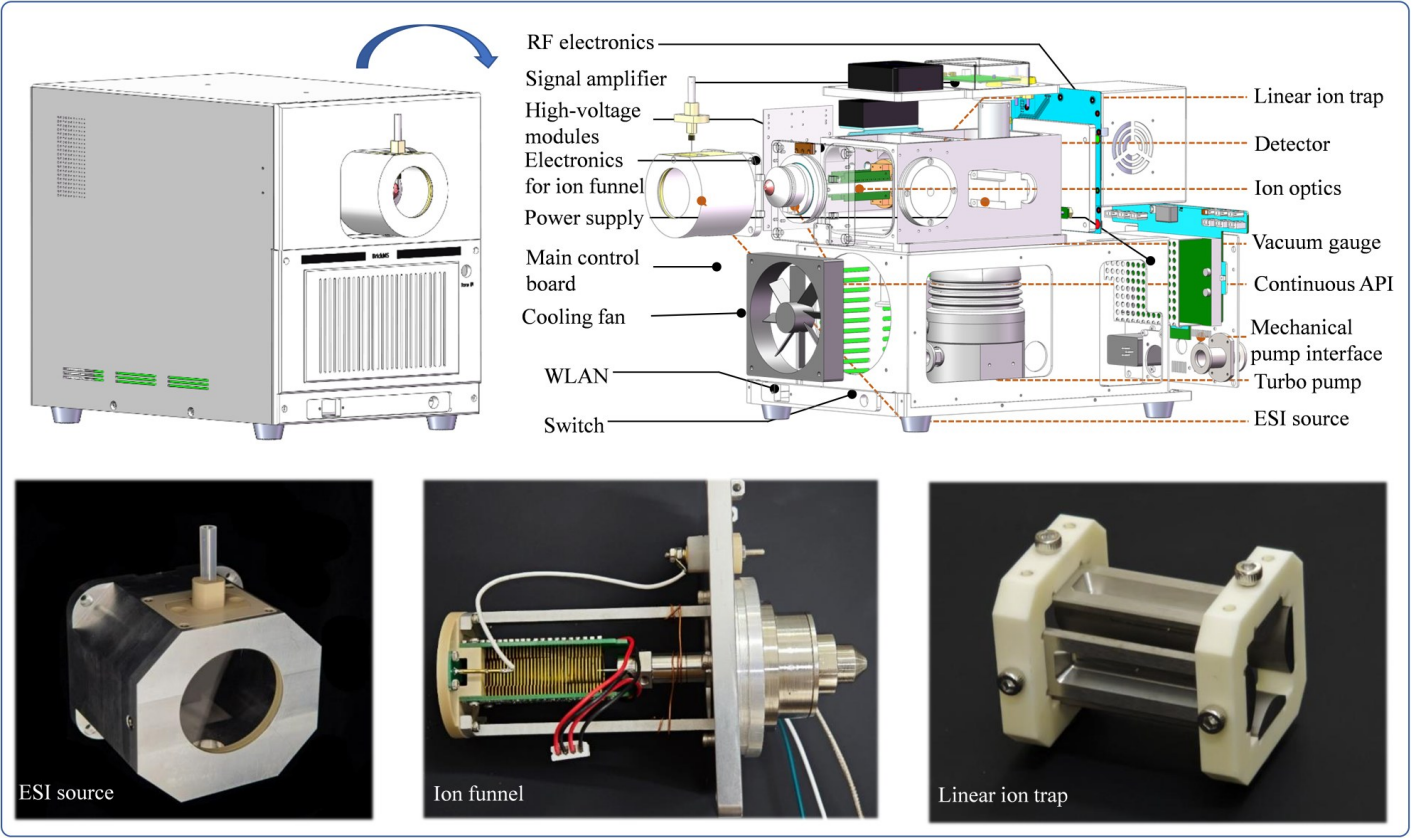
微型质谱仪详细构造图及核心组件实物图

高效原位离子化：将一次性采样装置与电喷雾离子化技术集成耦合，一次性采样嵌套管内堆叠集成有机滤膜、筛板、色谱填料等多功能组件，可以实现复杂基质样品的快速预处理，处理后的样品在重力、毛细管毛细作用和电喷雾驱动力多重作用下完成电喷雾离子化（图2“ESI source”）。

离子高效传输：离子传输是决定样品检测灵敏度的关键过程，是实现现场复杂体系及痕量物质检测的重点。采用一根垂直于离子漏斗轴向的不锈钢毛细管实现大气压进样，该接口可以避免中性分子及颗粒物沉积于后端的离子传输透镜，减小仪器污染，提高仪器分析复杂基质样品的灵敏度和鲁棒性。此外，该接口还将集成温控模块，实现进样管的快速升温和稳定控温，降低管内残留污染的同时可以增强电喷雾去溶剂化效果，提高离子化效率。离子漏斗由多电极片组成，是用于离子聚焦和传输的关键部件。基于对电场分布及离子运动状态的理论仿真模拟，离子漏斗综合运用了射频（RF）与直流（DC）电场的协同操控技术，通过优化电极几何结构与电场参数，实现离子的高效传输（图2“ion funnel”和“ion optics”）。

离子阱质量分析：离子被引入阱内后，在特定频率和幅度的射频电场作用下，不同质荷比的离子将呈现特定的稳定运动轨迹而被“囚禁”阱内。通过程序化地改变射频电压参数，可依次使不同*m/z*的离子运动失稳，从而按质荷比顺序“逐出”离子阱并到达检测器（图2“liner ion trap”）。

微型质谱仪系统集成：依托连续大气压接口小型质谱仪系统，基于两级真空腔体的小型差动真空系统，在一级真空腔体内集成小型离子漏斗传输系统，在二级真空腔体内整合离子阱质量分析器，用于样品采集及离子化的自主采样与复合离子源组件则集成于小型质谱仪大气压进样口前端。设计高精度、小型化的测控电路与软件系统，包括数据处理及控制系统、射频宽带高压放大模块、电荷信号放大调理模块、直流高压模块、高压电源模块等部分。最终，集成形成微型质谱仪原理样机。

### 1.2 仪器与试剂

Brick-L 2400微型质谱仪（昆山聂尔精密仪器有限公司），包含嵌套毛细管电喷雾离子源、数据传输线、Basic采集软件（Nier BrickMS Basic 1.3.2-Beta10）、Method筛库软件（Nier BrickMS Method 1.3.2-Beta10）、Analysis分析软件（Nier BrickMS Analysis 1.3.2-Beta10）等。Brick-L 2400微型质谱仪基于北京理工大学团队于2015年开发的质谱仪原理样机，并由昆山聂尔精密仪器有限公司完成集成转化。

甲醇、乙腈、甲酸均为色谱纯，购自美国Fisher Scientific公司；四乙基溴化铵、四丁基溴化铵、四己基溴化铵、四正辛基溴化铵、利血平标准品（纯度≥98%），购自上海阿拉丁生化科技股份有限公司；头孢呋辛酯片、中药丸戊己丸、依托考昔西药片均购自药店，唑吡坦药片产自赛诺菲（杭州）制药有限公司，经医院处方开具；实验用血为兔血，购自广东鸿泉生物，于‒20 ℃冰箱中保存备用。采血纸（Whatman 903）购自赛谱锐思（北京）科技有限公司。

### 1.3 实验内容

#### 1.3.1 样品准备

称取各标准品，以甲醇为溶剂，配制成质量浓度均为1 000 μg/mL的储备溶液，于4 ℃冰箱中保存。移取相应体积的标准品储备液，以含0.1%甲酸的甲醇-水（1∶1，体积比）混合溶液为溶剂，稀释成四乙基溴化铵、四丁基溴化铵、四己基溴化铵、四正辛基溴化铵、利血平质量浓度分别为3、1、0.5、1、5 μg/mL的标准品混合溶液，实验前现配。

头孢呋辛酯片、依托考昔西药片及中药丸戊己丸分别碾碎成粉末，备用。

将一片唑吡坦药片溶解于5 mL含0.1%甲酸的甲醇-水（1∶1，体积比）混合溶液，充分涡旋后取50 μL上清液加入1 mL兔血，混匀，制成血液模拟样本，将血液滴到采血纸上自然晾干2 h，打孔钳打3 mm直径干血斑。

#### 1.3.2 质谱分析过程

质谱分析按照开机-进样-测试-数据采集-关机的步骤完成。

开机：打开机械泵开关与质谱仪总开关，当气压达到设定分子泵启动阈值1.33 kPa时，分子泵自动开启，至气压达到设定软件可用阈值0.022 Pa且稳定后，可以正常使用。整个过程约5 min。

进样：移液器吸取50 μL标准品混合溶液注入嵌套管离子源，进行质量轴校准；西药片及中药丸粉末用经乙腈润湿的一次性洁净棉签轻轻蘸取放入装有萃取溶剂的嵌套管离子源中；干血斑置入嵌套管离子源，滴加200 μL萃取溶剂。萃取溶剂均为含0.1%甲酸的乙腈-水（7∶3，体积比）混合溶液。

测试：所有测试均采用正离子模式。一级质谱全扫描设置RF电压分别为300、500、800 V；选择离子扫描设置的目标离子分别为各样品相应目标成分的母离子：四己基溴化铵*m/z* 354.4、头孢呋辛酯*m/z* 533.1、依托考昔*m/z* 359.1、唑吡坦*m/z* 308.3；串联质谱分析在碰撞诱导解离（collision-induced dissociation，CID）模式下进行，碰撞电压分别设置为0.1~0.28 V和0.1~0.4 V；使用Method筛库软件进行中药丸主成分全自动筛查。

数据采集：待总离子流稳定后，Basic采集软件加载不同采集模式下的配置文件，勾选“High Voltage”，配置仪器各模块的高压信号参数，点击“Run”进行分析，采集谱图。

关机：测试结束后点击“关机”并确认，仪器自动关闭高压和分子泵，等待片刻，气压升高至266.64 Pa时，关闭机械泵开关和质谱仪总开关。

### 1.4 安全操作说明

针对仪器操作过程中可能误触到离子源、射频电源等高压信号的情况，实验过程中所有学生需要全程佩戴绝缘手套，并在操作前进行安全检查，确认所有电信号连接无误后再开始实验操作。实验操作人员须严格遵守真空泵异常急停操作流程：一旦出现真空度异常，第一时间在软件上关闭所有高压信号，然后关闭分子泵，待气压升至正常值后关闭机械泵。由于实验过程涉及挥发性有机试剂的样品配制等操作，所以需要实验人员全程穿着实验服并佩戴口罩。

## 2 结果与讨论

### 2.1 质量轴校准

为了确保质谱仪准确测量离子质荷比，在测试前需进行质量轴校准。其原理是通过已知不同质量的校准品混合物，对质谱仪的质量轴进行校正，以确保测量值与理论值一致。校准曲线方程为*Y*=0.215*X*+5.358，其中*Y*为质荷比，*X*为RF电压值；线性相关系数（*R* ^2^）=0.999 5。校准通过后，结果自动保存，仪器可用于后续实验。如有更大质荷比的样品需要测试，可添加相应范围的校准品进行高质量段的校正，根据需求可以拓展至*m/z* 2 000。

### 2.2 离子漏斗射频电压对离子传输效率的影响

用Analysis分析软件对比分析不同RF电压下的标准品混合溶液的一级质谱谱图，结果见图3。RF电压为300 V时，离子*m/z* 130.1强度约为1 000，随着RF电压提高强度逐渐下降；RF超过800 V，该离子无法测出。*m/z* 242.3的离子在RF值为500 V时，信号强度较高，升至800 V后信号则明显减弱。*m/z* 466.5和*m/z* 609.7的离子随着RF电压升高，信号强度均呈明显增长趋势。这是因为施加在线性离子阱上的RF电压用于约束离子在阱内的径向运动，较低质量的离子在较低RF电压下易被释放，而较高质量的离子需要更高的RF电压才能被释放，通过改变RF电压的幅度，可以实现对不同质量离子的选择性捕获和释放。同时，RF电压的变化会影响离子在离子阱中的运动稳定性。当RF电压升高时，高质荷比离子变得不稳定并从阱中射出，从而影响离子的出峰，因此通过调节RF电压可以改变谱图中不同质荷比离子的出峰。在确保仪器安全的前提下，学生可自行调整RF电压参数，观察不同参数对实验结果的影响。

**图3:**
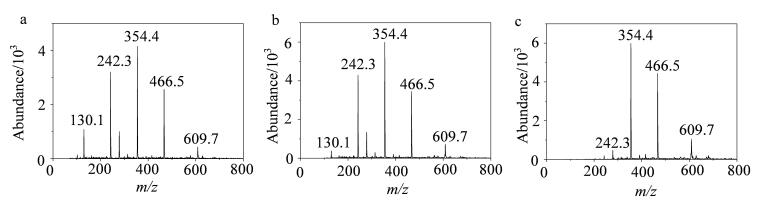
不同RF电压条件下标准品混合溶液的全扫描质谱图

### 2.3 串联质谱分析

#### 2.3.1 一级质谱分析

离子阱质谱仪中的离子孤立是一个至关重要的步骤，它是整个MS/MS或MS *^n^* 分析流程的起点和核心环节。离子孤立是指在离子阱内，从同时存在的众多离子中，选择性地保留一个特定质荷比或一个狭窄质荷比范围的离子，同时高效地排除所有其他不需要的离子（包括背景离子、化学噪声、加合物离子等）的过程。*m/z* 354.4离子的选择离子扫描谱图见图4a。设置目标母离子质荷比、孤立缺口宽度、偏移等值，*m/z* 354.4 的离子被孤立出来，其他质量的离子被排除，孤立参数设置影响到目标离子的孤立效果。

**图4:**
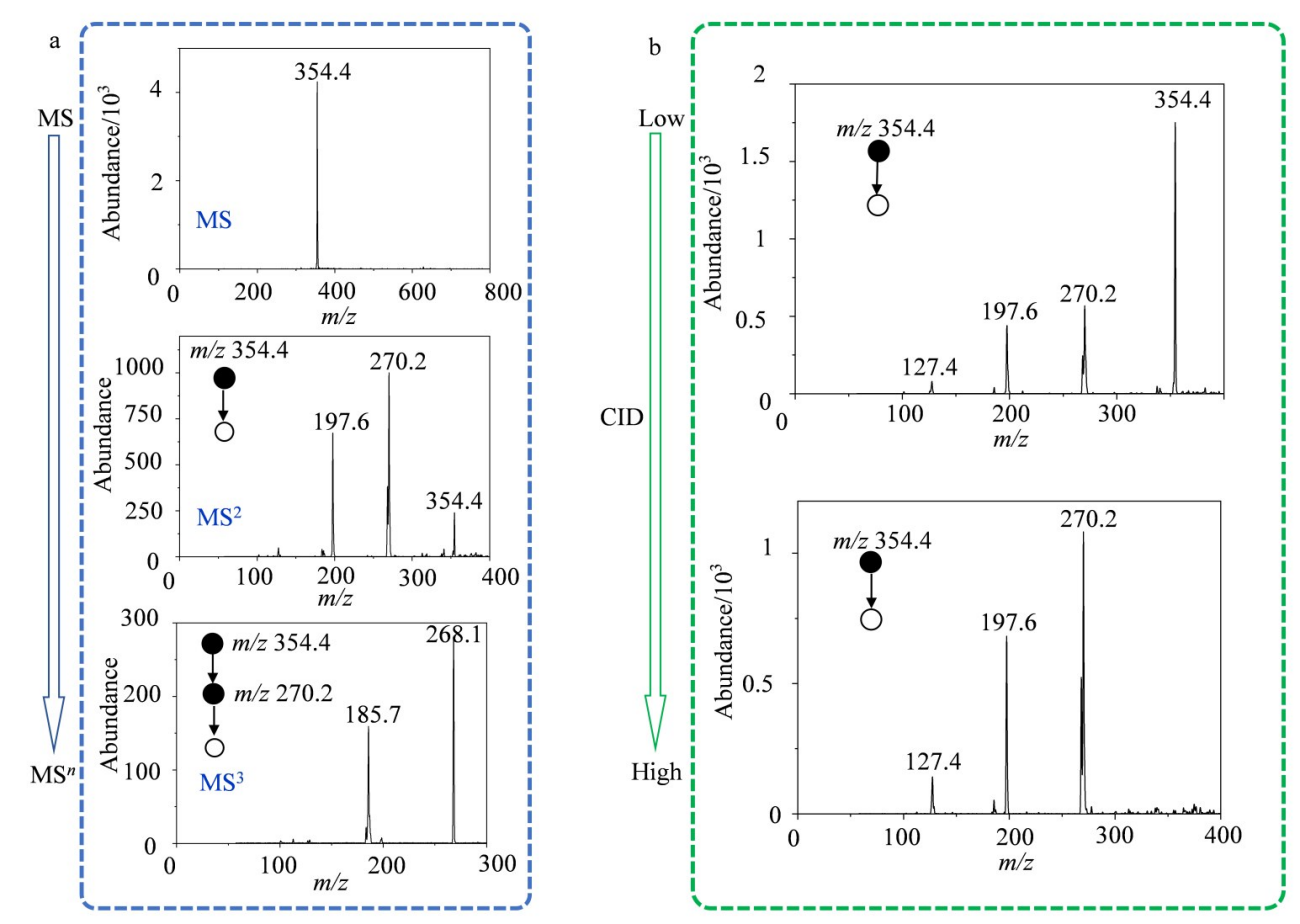
标准品混合溶液的串联质谱分析（以*m/z* 354.4离子为例）

#### 2.3.2 二级质谱分析

*m/z* 354.4母离子的二级串联质谱图见图4a。谱图中母离子在离子阱内孤立，通过施加碰撞能量，离子发生解离产生碎片离子，生成的碎片离子仍然被离子阱捕获，出现了两个丰度较高的*m/z*为197.6和270.2的碎片离子。

#### 2.3.3 三级质谱分析

选择*m/z* 270.2碎片离子作为母离子进一步碎裂，获得该离子的碎片离子，得到*m/z* 354.4离子的三级质谱图，即*m/z* 185.7碎片离子，见图4a。在此基础上，学生可自行选择其他母离子及相应的碎片离子，采集更多的谱图进一步理解串联质谱分析的原理，仅一个离子阱质量分析器就实现了时间上的串联。

#### 2.3.4 碎裂效率表征

针对*m/z* 354.4母离子分别设置两个CID电压范围。如图4b所示，设置为0.1~0.28 V时，*m/z* 354.4母离子仅有一部分被打碎，生成了*m/z* 197.6和*m/z* 270.2的碎片离子；设置为0.1~0.4 V时，所获得的二级谱图中不再有*m/z* 354.4离子，母离子被全部打碎，同时碎片离子的丰度也增大。说明对目标离子进行串联质谱分析时，需要调节合适的CID电压值才能保证目标离子能够被有效碎裂，从而获得较多的碎片离子信息和较高的碎片丰度。

### 2.4 西药片快速分析

头孢呋辛酯片、依托考昔两种药片的全扫描、选择离子扫描和串联质谱分析结果见图5。头孢呋辛酯母离子的质荷比为533.1（图5a、5b），丰度较高的碎片离子*m/z*为447.3和489.3（图5c），并推断*m/z* 447.3离子所对应的结构是头孢呋辛酯母离子脱去乙酸乙酯基团后得到的碎片离子；依托考昔的*m/z*为359.1（图5d~e），丰度较高的碎片离子*m/z*为280.1（图5f），该离子则是依托考昔母离子脱去苯环上甲磺酰基后得到的碎片离子。在此基础上，可引导学生继续根据化合物结构和碎片的质荷比，进一步推断其他碎片所对应的结构及相应裂解方式，理解串联质谱分析在未知样品结构定性方面的应用。

**图5:**
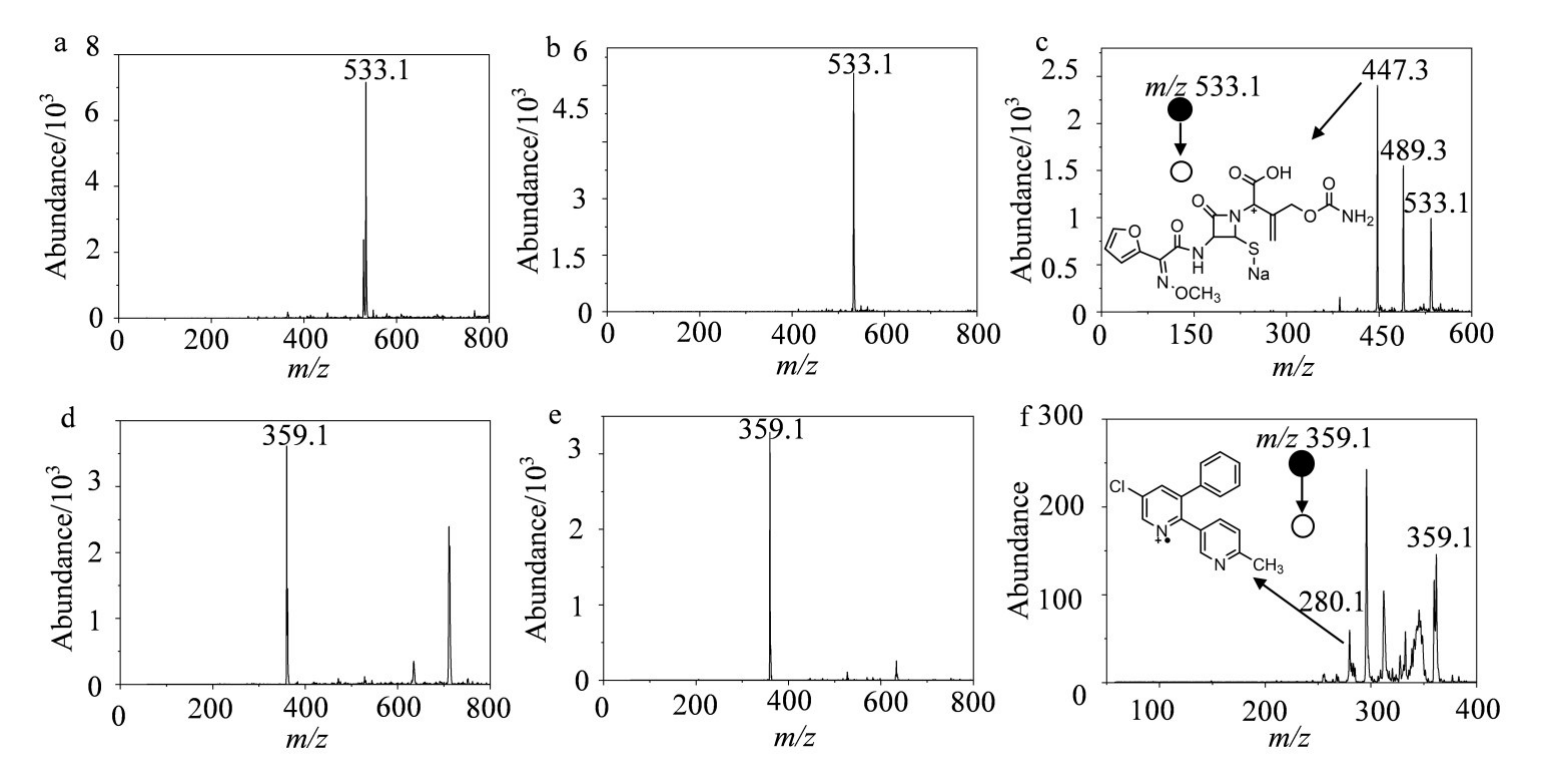
西药片头孢呋辛酯和依托考昔的质谱分析

### 2.5 全血中唑吡坦药物的快速检测

利用质谱技术对血液中的药物及其代谢产物进行定性和定量分析的方法，在临床治疗药物监测（therapeutic drug monitoring， TDM）^［27，28］^和毒理学检测^［29-31］^等领域具有重要应用。唑吡坦血药干血斑的全扫描、选择离子扫描和串联质谱二级谱图见图6。不同于标准品混合溶液和西药片，干血斑中药物目标离子的质谱信号响应会受血液中蛋白、脂肪、盐等基质的影响，使用大型质谱仪进样前需要对样品进行复杂的前处理。微型质谱仪快速检测结果显示，唑吡坦母离子的*m/z*为308.3（图6a、6b），丰度较高的碎片离子*m/z*为235.3和264.4（图6c）。干血斑和萃取溶剂实现了在线除杂的功能，进而快速准确检测到血液中的药物。

**图6:**
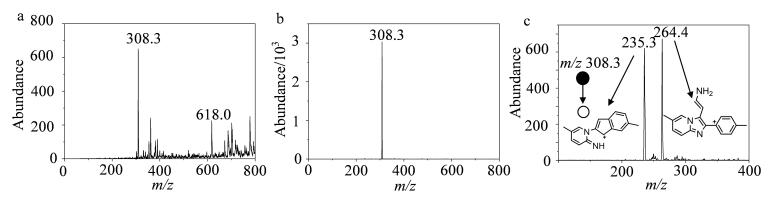
血液中唑吡坦的质谱分析

### 2.6 中药丸中多种生物碱成分的质谱谱库快速筛查

质谱谱库筛查是一种基于质谱数据的分析方法，通过将实验获得的质谱数据与预先经标准品建立的标准谱库进行比对^［32-34］^，从而识别和鉴定样品中的化合物，这种方法广泛应用于代谢组学、蛋白质组学、药物分析等领域。用户通过采集标准品自建谱库，可实现特定研究领域化合物的快速筛查。基于已建中药谱库，利用Method筛库软件，以母离子和二级碎片离子同时匹配为依据，中药戊己丸筛查的结果匹配度较高的主成分共有7种，见表1。

**表1:** 戊己丸中生物碱成分的筛查结果

Compound	Match degree/%
Coptisine （黄连碱）	100
Berberi （小檗碱）	89
Epiberberine （表小檗碱）	87
Evocarpine （吴茱萸卡品碱）	85
Dehydroevodiamine （去氢吴茱萸碱）	83
Palmatine （巴马汀）	76
Dihydroevocarpine （二氢吴茱萸卡品碱）	74

## 3 教学实践与反思

### 3.1 教学安排

本实验是仪器分析课程中质谱这一部分的教学内容，也可作为开放型实验课程面向化学、生物、环境科学、电子、机械等多专业的学生开展。考虑到涉及的教学内容深入、涉及多学科知识，建议学生5~6人为一组，协同合作，设计实验方案并全员参与完成。课程开展包含课前预习、课中实操、课后总结、撰写报告几部分，实验学时建议为12学时。

#### 3.1.1 课前预习

学生自行通过网络课程或相关文献，预习基础理论知识。实验课程开始前，教师采用动画、视频等方式，详细讲解仪器的原理和关键部件作用。

#### 3.1.2 课中实操

该部分内容由学生动手操作完成。

仪器拆装：在仪器不通电情况下，拆去外壳展示完整构造，认识装配完整的微型质谱仪内部各部件排列，包括真空系统、进样系统、传输组件、质量分析组件、检测器等；经教师指导，学生之间互相合作拆下离子源、离子漏斗、离子阱等关键配件认真观察，教师讲解每个配件的工作原理及各配件如何配合实现样品的离子化、传输及采集。再由学生将拆下的配件按照原状态装配回仪器，由教师确保装配手法正确、位置无误。该部分4学时。

数据采集：仪器通电开机，打开采集软件，教师向学生详细讲解各参数含义及调节方法，以方便学生在实验过程中自主调节参数，观察实验现象。指导学生进样。首先进行质量轴校准，保持持续进样，学生按照实验内容进行各模式下数据采集，调节参数获取多种条件下的采集结果并保存。标准品混合溶液数据采集过后，进行实际样品的测试，测试顺序为先采集两种西药片的质谱数据，再对干血斑进行血液中药物的分析，最后进行中药丸多组分的谱库筛查。所有数据采集完毕后，教师指导学生用分析软件分析并导出各数据，以便于后续进一步分析和实验报告的撰写。该部分6学时。

课后总结：组织学生就实验中出现的问题及现象提问、复盘，必要时可重复相应实验。听取学生上课感受，争取获得每一位学生的反馈。该部分2学时。

### 3.2 教学反思

该课程目前已在线下教学中实施一次，教学实践表明，这种“结构-原理-应用”深度融合的教学模式，不仅有效贯通了从理论学习到实践应用的完整知识链条，更能通过真实科研课题的导入，显著提升学生的创新思维和解决复杂分析问题的能力，为分析仪器类课程的改革提供了创新范式。后续教学可进行以下优化：（1）在仪器原理认识及拆装阶段，增加外部辅助测试设备，直观体现各配件的功能；（2）结合生活科研扩大样品种类，例如可引入临床样品，进行血液浓度的监测，引入生活中的非法添加如摇头丸等加深实验的深度和广度。

## 4 结语

本文基于微型质谱仪的详细构造原理和多种功能，依据教学目标设计了内容丰富、全面的教学实验，可用于高年级本科生和研究生的仪器分析实践课程。该教学以仪器各部分组成及作用为基础，让学生深入认识质谱仪的原理；实验从质量轴校准、全扫描、选择离子扫描、串联质谱数据采集切入，展示不同模式下质谱分析的过程；结合参数调节对结果的影响，进一步加深学生对质谱原理及运用的理解。在此基础上，进行常见药物和血液中药物的检测，实现真实样品在微型质谱仪上的快速分析，并拓展至质谱仪在临床治疗和毒理学检测等领域的实际应用。最后，依据微型质谱仪自建谱库，完成中药丸中主成分的快速筛查，丰富学生关于质谱应用的全面知识。教学内容具体深入、层次分明、重点突出，能够大幅度提升学生的理论和实践素养；实验样品容易获取和储存，试剂简单安全，适合在高校实验室中进行。该教学实验不仅有助于显著提升学生的综合素质，还能够全方位地培养他们的多维思考能力和实验操作技能。学生在理论与实践的结合中，深入理解复杂的科学概念，锻炼解决实际问题的能力。这种综合性的学习体验，为学生未来踏入科研领域或从事相关应用工作奠定了坚实的基础，并提供宝贵的启发和指导，有助于培养他们卓越的创新思维和实践能力。
